# Miscellaneous Cases

**Published:** 1828-08

**Authors:** 


### MISCELLANEOUS CASES.

### *Cases of Contraction of the Pulmonary Artery, treated at* St. Thomas's Hospital.

Owen Sweeny, aged forty-nine, had been ill five years. When admitted, he had ascites, anasarca of the legs, a quick and rapid pulse, dyspnœa, and palpitation; but could lie down. The palpitation and dyspnœa had existed a year. The jugulars and other veins of the neck were distended to a great degree. On applying the stethoscope to the right side of the heart, or upon the sternum, a whizzing sound (*bruit de soufflet*) was heard; and it was ascertained, by feeling the pulse, that this sound was synchronous with the contraction of the ventricles.

The principal post-mortem appearances were as follow: The pericardium was adherent to the heart, and contained some portions of cartilage; there was a cartilaginous body in the substance of the wall of the right ventricle, where the pulmonary artery leaves it, and the artery was contracted in size to that of the brachial, there and for some inches beyond.

These particulars have been kindly furnished by Dr. Elliotson, and they prove that his diagnosis was perfectly correct.

Dr. E. had not met with another case of the same kind until within the last two months, when a patient came to the hospital with symptoms so much resembling those of the former case, that he immediately declared that it arose from the same cause.

The man's name was Crawley, his age sixty, and he had been out of health some months. The general symptoms were orthopnœa, anasarca of the arms, thighs, and legs; considerably increased action of the carotids and radials, and distention of the veins of the neck, with tenderness of the epigastrium. The stethoscope, as in the former case, gave the only certain indication of the cause of the disease. On applying it to the upper part of the sternum, a loud and distinct *bruit de soufflet* was heard, at the moment when the ventricles contracted, proving that the obstruction must be at the outlet of one of those cavities, while the situation in which the noise was heard, and the distention of the veins, pointed out the right as the one implicated. The only material differences between the two cases were the circumstance that, in the former, the patient could lie down, while the latter could not, and the increased action of the carotid and radial arteries in the latter. These did not attract much attention at the time, and they most probably arose from a very different cause from that which produced the other symptoms. What this cause was will appear in the sequel.

*Sectio cadaveris.*—This was performed under very unfavorable circumstances, being done almost by stealth, in the patient's house, and when the body was in a very advanced state of decomposition. In consequence, a very minute examination could not be made, but the heart itself was brought away.

The pericardium was adherent to the surface of the heart in every part; the heart itself was enlarged to twice its natural size, and its substance was very much softened, and so changed in texture as almost to have lost its fibrous appearance. A part of this change might be owing to the decomposition, but certainly not all of it. The walls of the cavities were thickened, but not in proportion to the increase in size of the whole heart; the cavities themselves, and especially those on the right side, being much dilated. At the origin of the pulmonary artery, a fibro-cartilaginous structure was found, as large as a small egg, and almost surrounding the artery; which was, in consequence, so much diminished in calibre, that it would scarcely admit the little finger: beyond, the artery retained its usual size. Here, then, was precisely the morbid change which had been foretold, and another proof that the stethoscope is not quite so useless an instrument as some suppose it to be. But another most unexpected disease was found in the chest: a very large aneurism of the aorta, which had burst before the body was opened, and probably before the patient's death, as the blood with which the back part of the chest was filled had coagulated. This was not looked for; but might it not have been so? Was there not, at least, one symptom of it? It has been already stated, in the account of the symptoms, that there was increased action of the carotid and radial arteries, and that the patient could not breathe in the recumbent posture: both these symptoms were present, in a remarkable degree, in the case of aneurism of the aorta described in the Gazette a few weeks since. The latter is a usually described symptom of the disease: may we not conclude that the former also, although it has not hitherto been mentioned as a symptom of aneurism of the aorta, is yet one, at least, of the aneurism by dilatation, which both these cases were? Two cases are scarcely sufficient on which to build an opinion; and it appears almost incredible that such a remarkable symptom should have been overlooked, if it had existed in other cases.

The obliteration of the cavity in which the heart naturally moves, by the adhesion of the two surfaces of the pericardium, was a very remarkable point of resemblance in both the cases described in this report. It must materially have contributed to produce the symptoms; and Dr. Elliotson is inclined to think that adhesion of the loose pericardium, from inflammation, is a very common commencement of many diseases of the heart, as well as of that above described. It will be remembered that in the first case (that of Sweeny,) the pericardium was beset with pieces of cartilage, resembling that which produced the obstruction.

It may, perhaps, be said, that when there was so much other disease, and especially in the latter case, (aneurism of the aorta,) it is unfair to attribute the symptoms to the obstruction of the pulmonary artery. But, first, such an obstruction could not exist without producing some very marked effects.

Secondly, the main symptoms were precisely those which diminution of the outlet of the artery would produce.

Thirdly, LAENNEC asserts—and subsequent pathological observation has confirmed the truth of the assertion,—that the stethoscope is incapable of giving any certain indication of the existence of aneurism of the aorta, and that it is continually found without having been suspected.

Fourthly, whatever may be the real symptoms of such an aneurism, neither it nor adhesion of the pericardium have ever been known to produce a ventricular *bruit de soufflet* confined to the right side.*

---

*Case of Extra-Uterine Fœtation, in which the Fœtus remained in the Abdomen forty years.* Communicated by HENRY LEE HEISKELL, M.D. of Winchester, Va.

THE following case occurred in the poor-house of the parish of Frederick, in Virginia.

Venus, alias Venus Collins, a coloured woman, emancipated in November 1795, by Sarah Zane, deceased, late of the city of Philadelphia, became pregnant with her seventh child, which she bore until her death, which occurred in the summer of 1825. As near as could be ascertained by a reference to the records of the clerk's office, and the statements of her fellow servants, (one of whom was her daughter-in-law,) she was from seventy to seventy-five years of age, and carried the fœtus forty years. During this period, and in particular the latter part of it, she enjoyed remarkably good health for one in her situation, being only occasionally incommoded by a sense of weight and bearing down in her right side, which was sometimes accompanied with slight pain. In the early part of her pregnancy, she had hydropic effusions, for which (as well as I could ascertain) she underwent the operation of paracentesis abdominis. She had no show of the menses after this period, nor did afterwards conceive again. For several years before her death, the infirmities attendant on old age, and the difficulties of providing for herself, rendered her removal to the Poor-house necessary, where she remained until her death, which happened in consequence of an attack of dysentery.

A post-mortem examination of her body was conducted by Drs. A. S. BALDWIN and HOLLIDAY, in presence of several medical students. On making a crucial incision through the parietes of the abdomen, and turning back the flaps, a large bony tumor was found in the lower part of the epigastric region, inclining rather to the right side, and firmly agglutinated in front to the parietes of

* Medical Gazette.

the abdomen, and behind to the small intestines. The only morbid appearances of the viscera presenting, were a diminished size of the uterus, and an obliteration of the fallopian tubes: the ovaria were not to be found.

The tumor itself was of an oblong form, which, when removed from its attachments, weighed four pounds and six ounces. The envelope formed a perfect bony hermetically sealed sac, on all sides, but rather thin at the part corresponding to the anus; for, when considerable pressure was made in the direction of its short diameter, a few drops of dark fluid made its way through the covering.

The substance of the sac or covering was of an ossific nature, of a dirty-white or cream colour, varying from two to three lines in thickness; so resisting, that it required not only a strong knife for its division, but also a very considerable degree of strength.

On removing the sac, which had formed adhesions to several parts of the fœtus, particularly the superior part of the right thigh, a fœtus, perfect in its form and configuration, was presented, having apparently gone the full period of utero-gestation. Its position in the sac exactly resembled that of a fœtus in utero; having the chin resting upon the chest in such a manner that the face looked towards the left side; the trunk was incurvated, the legs bent upon the thighs, the thighs upon the pelvis and abdomen, the feet crossed, and the arms folded between the head and knees. Owing to the firm pressure of the sac, the abdomen and lower part of the chest received the impression of the arms and thighs; and the latter, in turn, from the same cause, were somewhat flattened.

The weight of the fœtus, divested of its covering, was three pounds and three-quarters, and measured, in its contracted state, eleven inches and one-half in length. So faultless was every limb and feature, (with the exception above stated,) that no one of them presented an exception worthy of special remark. The general aspect, however, of the fœtus bore evident marks of *age*, (if the remark might not be considered a contradiction.) The muscles and integuments were firmer and more consistent than in the natural state, and the latter were very generally ossified, except those portions which were covered by the foldings of the arms and thighs: consequently the integuments which partook of the ossific character had a decided preponderance over the parts which did not take on a change of structure. The pericranium was entirely in an ossified state, over which some traces of hair were discernible, and the remains of the eyelashes were distinctly perceptible.

On examining the contents of the cranium, thorax, and abdomen, the following appearances were noticed: The brain was a soft pulpy mass of an ash colour, presenting nothing very remarkable in its appearance. The contents of the thorax and abdomen were in a singular state of preservation, as perfect as those of a stillborn child; nothing of decay or putrefaction could be discovered in any portion of them. The meconium exhibited the usual dark appearance and consistence. The tongue was firm and ash-coloured; and the nails of the fingers and toes perfect.

No traces of umbilical cord or placenta could be discovered.*

---

### *Case of Sudden Death connected with Organic Disease of the Heart.*

By Harper Walton, M.D.

Partrick M'Claskey, æt. fifty-six years, was admitted into the Philadelphia Alms-house Infirmary, several months previously to his death, which occurred suddenly on the 7th of March, 1827. During the month of December, 1826, he was observed to labour under considerable dyspnœa and irritating cough. His face exhibited a livid complexion, and this appeared to be produced by a congestion of the capillary vessels with imperfectly decarbonated blood. He was distressed with feelings of anxiety, and entertained apprehensions of death. This patient had frequently been observed to have a pulse remarkable for its slowness and irregularity. On the 23d of January, 1827, I examined this attentively, and found it full and voluminous; beating, also, thirty-six times in one minute precisely, with equal intervals of time between the pulsations. The venous complexion of the face, the paroxysmal dyspnœa, with a state of general debility, continued till the hour of his death.

The antispasmodic remedies, especially the Lac Assafœtidæ, and a combination of Hoffman's anodyne with laudanum, in small and repeated doses, were found useful palliatives of the cough and difficult respiration. Mild laxatives, with a light and easily digestible diet, were administered throughout his disease, with advantage.

On the 7th of March, about ten A.M., I was requested to see him immediately, as he had been suddenly seized with "a fit," resembling apoplexy, only a few minutes before. On my arrival at his bedside, I found him perfectly motionless; his lips livid; his face pallid; and, in short, every symptom denoting death. As the jugular veins were very turgid with blood, I immediately opened one of them, and drew a considerable quantity of blood, which, however, produced no kind of effect. I learned from those who had witnessed his death, that he had eaten his breakfast in the morning as usual, and that he had spoken to an assistant of the ward on some familiar topic, about a quarter of an hour before his death. They also stated that the attack was very sudden, that it resembled a fit, in which there were violent muscular exertions, and that it lasted about ten or fifteen minutes.

Twenty-four hours after death, assisted by my friend Dr. Ashmead, I made the post-mortem examination. Nearly the

* American Journal of the Medical Sciences.

whole of the exterior surface of the lungs was found united to the parietes of the thorax, by long-established adhesions of the pleuræ. The heart was found unusually large, soft, and flaccid. The right auricle was found much enlarged; its parietes very thin, and in some of its parts resembling a translucent membrane. The orifices of the coronary veins were considerably dilated. The tricuspid valve had a reddish colour, with less transparency than what is usually observed. The right ventricle was also dilated. Both of the left cavities of the heart were very much enlarged, and the mitral valve which intervenes appeared thickened from the effect of diseased action. The lining membrane of the left ventricle was found, in some of its portions, thickened and opaque; and near to the junction of this cavity with the aorta, there was a deposit of osseous matter.

Upon the surface of the semilunar valves, at the origin of the aorta, there were also marks of ossification, and a few points of ossific formation were discovered upon the lining membrane of the great artery at its curve.

Immediately after leaving the heart, the aorta was found dilated to twice its natural size, and those portions of it situated about the origin of the innominata, left carotid and left subclavian arteries, from their thinness were very easily torn.

The stomach presented a natural appearance, but the texture of its mucous coat was softer than what is usually found in one perfectly free from disease.

On examination of the brain, a considerable quantity of effused serum was found between the dura mater and the arachnoid membrane. The lateral ventricles were much distended with this fluid. The substance of the cerebrum was unusually firm; that of the cerebellum uncommonly soft. Numerous red points, from congested vessels, appeared when incisions were made through the medullary portion of the cerebrum.

Was apoplexy in this case the immediate cause of death? It has been remarked by a physician of high authority, that "the predisposition to apoplexy is probably laid in all those causes which produce a decay in the moving powers of the circulation, in organic feebleness," &c. &c. Causes such as have been described had evidently, for a considerable period, produced that disordered state of the circulation which would result from a "decay in its moving powers." They increased, moreover, in force, up to the period of the patient's death. The venous colour of the capillaries of the skin,—the turgescent condition of the venous system during life,—the extreme slowness of the pulsations of the heart,—the dyspnœa, &c. furnished proof sufficient of a greatly obstructed circulation. In this state of repletion the venous system of the brain must have shared largely; and when we take into view the extreme vascularity of that organ, we can readily account for the sudden and fatal injury which it sustained.

According to ABERCROMBIE, apoplexy depends "on an interruption of the course of blood from the brain," which in effect points to congestion of the vascular system of this organ and its meninges as the immediate cause of the disease.

By SERRES it has been denied that compression of the brain is a cause of apoplexy. By this position, congestion, as a cause of apoplexy, is also denied; for compression is an obvious result of a congestion of the vascular system of the encephalon. The opinion of Serres was founded on experiments made upon dogs, in which he failed to produce apoplexy, either by compression of the brain artificially, or by producing effusions of blood within the substance of the cerebrum. In man, however, compression of the brain, by depression of the cranial bones, or by effusions within its substance, is a very common occurrence; and with it, also, apoplectic symptoms have been strongly marked. In performing experiments upon the brain of animals, in order to ascertain the effects of its compression, it appears to me impossible that there can be a condition produced by art similar to that which exists in cases of apoplexy the natural course of disease. Artificial compression cannot be made to affect the brain beyond a certain degree; that degree must be partial, though it may be great; but that produced by congestion or distention of the whole cerebral vascular system must evidently be universal, and consequently more fatal in its effects.

By recurring to the description of appearances presented by the examination of the brain, further proof of fatal injury, by congestion of this organ, will be immediately evident. There could not, indeed, be discovered any effusions of blood into the cerebrum, cerebellum, or cranial cavity, or any rupture of blood-vessels; but these are by no means necessary attendants, since observation has proved that apoplexy may happen without any of these.

In the Dublin Hospital Reports for 1827 will be found an interesting paper, by R. ADAMS, which contains cases illustrative of apoplexy connected with organic changes affecting the structure of the heart.*

* Ibid.

